# Self-Reported Burden and Health-Related Quality of Life in Acute Kidney Injury Survivors Compared with Patients with Advanced CKD

**DOI:** 10.34067/KID.0000000707

**Published:** 2025-01-17

**Authors:** Félix Huang, Isabelle Ethier, Isabelle Vaillant, Samuel A. Silver, Jean-Maxime Côté

**Affiliations:** 1 Faculty of Medicine, Université de Montréal, Montréal, Québec, Canada; 2 Division of Nephrology, Centre hospitalier de l’Université de Montréal, Montréal, Québec, Canada; 3 Centre de recherche, Centre hospitalier de l’Université de Montréal, Montréal, Québec, Canada; 4 Division of Nephrology, Kingston Health Sciences Center, Queen's University, Kingston, Ontario, Canada

**Keywords:** AKI, CKD, cohort studies, health status, patient self-assessment, patient-centered care, quality of life

## Abstract

**Key Points:**

- Patients recently discharged after a severe AKI episode have a low health-related quality of life.
- This self-reported quality of life is comparable with that of people living with advanced CKD.

**Background:**

Survivors of severe AKI are at risk of increased morbidity. There are limited data of the quality of life (QOL) of patients who recently have had an AKI episode. The purpose of this study was to explore the health-related QOL of severe AKI survivors when compared with patients living with advanced CKD.

**Methods:**

A prospective observational cohort study of severe AKI survivors who attended follow-up in a specialized post-AKI clinic was compared with a cohort of patients with advanced stage 4 or 5 CKD followed in a dedicated nephrology clinic. Self-reported health-related QOL was determined with the Treatment Burden Questionnaire (TBQ) and compared between both groups.

**Results:**

A total of 100 participants were included in this analysis. As opposed to participants with CKD, AKI survivors were younger (median age: 63 [52–72] versus 72 [62–79] years) and had lower comorbidities (median Charlson index: 5 [2–7] versus 8 [6–9]) and higher eGFR at follow-up (median: 60 [40–82] versus 15 [12–20]). The overall QOL reported by AKI survivors was not statistically different from participants with advanced CKD (median TBQ score: 19.0 [6.5–43.8] versus 25 [12.8–43.0]), *P* = 0.45). The only domain where a significant difference was observed between the two cohorts was dietary restrictions (*P* = 0.037). A negative correlation between age and TBQ score was observed (Spearman's rank: −0.23 [*P* = 0.02]), especially in participants with CKD (−0.51 [*P* = 0.001]), meaning older participants reported a lower effect on health-related QOL, and between eGFR and TBQ (−0.20 [*P* = 0.049]), meaning participants with lower eGFR reported lower health-related QOL. No correlation was observed for hospital length of stay, burden of medication, or follow-up duration since hospital discharge.

**Conclusions:**

This study showed that health-related QOL of patients recently discharged after a severe AKI episode is comparable with the low QOL reported by patients living with advanced CKD. How to integrate that information into clinical practice when offering post-AKI care requires further research.

## Visual Abstract

**Figure:**
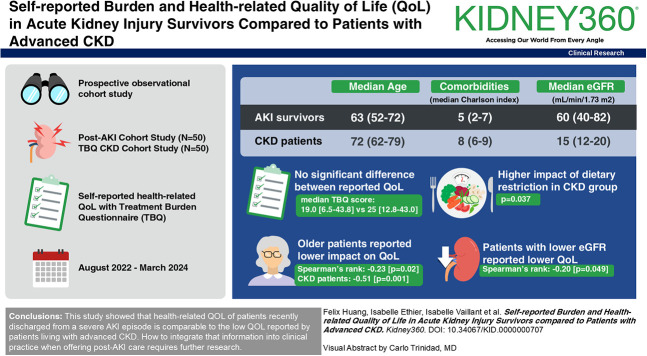

## Introduction

Patients with CKD experience a reduced health-related quality of life (QOL) compared with the general population.^1,2^ In advanced CKD, including ESKD, low health-related QOL is associated with higher mortality and risk of hospitalization.^1,3^ The effect of impaired kidney function on overall QOL is sometimes profound, particularly among those with advanced CKD and comorbidities, which has been compared with patients with cancer or heart failure.^4,5^ The symptom burden and overall QOL of patients living with CKD have been extensively studied^6^ and is now part of interdisciplinary care we routinely offer. There is also some evidence that the self-reported QOL of patients who have had an AKI episode may be lower than the general population.^7–10^

AKI is associated with increased morbidity, mortality, and cost of care.^11–13^ There has been recent awareness of the importance of offering dedicated post-AKI care. However, such care has mainly focused on residual kidney function and proteinuria assessment and potential nephroprotective interventions at follow-up in an attempt to mitigate cardiovascular events, as well as the AKI-to-CKD transition in AKI survivors.^14,15^ As with all chronic conditions requiring longitudinal care, there has been an increasing move toward a patient-oriented approach, from the hospital stay to the community follow-up. There are currently substantial resources available to support people living with CKD through various organizations^16^ and clinical settings (*e.g*., structured multidisciplinary care in advanced CKD clinics).^17^ However, when considering post-AKI care, especially in severe AKI survivors, there is a paucity of data on the self-reported QOL and burden, as well as a substantial lack of formal resources specific to these patients having recently survived complex and prolonged hospitalization.

To better inform the implementation of effective patient-oriented care and provide relevant resources in newly created post-AKI clinics, the objective of this study was to explore the health-related QOL of severe AKI survivors when compared with patients living with advanced CKD using a standardized questionnaire. Our initial hypothesis was that AKI survivors have a lower perceived burden of disease than people living with CKD because their condition is often considered to be resolved. However, certain aspects of health-related QOL, such as the burden of doctor appointments and laboratory tests, may be affected by this acute episode requiring medical follow-up after hospital discharge.

## Methods

### Design and Population

This prospective observational study was conducted at the Centre hospitalier de l’Université de Montréal (CHUM), a large academic health center in Canada. It consisted of two independent prospective cohort studies: the Post-AKI Cohort Study and the Treatment Burden Questionnaire (TBQ) CKD Cohort Study. The health-related QOL was evaluated through the TBQ, a standardized self-administered questionnaire.^18^

The Post-AKI Cohort Study is an observational, longitudinal, and multicenter cohort study active since August 2022. This study integrates various research objectives, including reports of health-related QOL outcomes. Its inclusion criteria are being referred to the dedicated local post-AKI nephrology clinic, recent hospitalization (<90 days) complicated with a moderate-to-severe AKI episode (Kidney Disease Improving Global Outcomes [KDIGO] AKI stage 2–3), and capacity to give direct consent. Patients who had solid-organ transplant and patients with preexisting stage 5 CKD, those on ongoing KRT (any modality), and non-French or English speakers were excluded. Patients with active GN or early reversible AKI were also excluded. At the initial post-AKI outpatient visit (8–12 weeks after discharge), the patient is approached by an independent research coordinator for consent. After obtaining consent and urine collection, paper-format questionnaires (including TBQ) are completed before the medical visit to minimize the risk of bias. Only participants of the Post-AKI Cohort Study from the CHUM were included in the present study.

The TBQ CKD Cohort Study is an independent, observational, cross-sectional cohort study. The main objective of this study was to report health-related QOL outcomes using the TBQ in outpatients living with advanced CKD. Recruitment occurred between June and August 2023. Its inclusion criteria were ongoing follow-up at the CHUM renal protection clinic and KDIGO stage ≥4 CKD. Non-French or English speakers and patients undergoing active KRT (any modality) were excluded. After eligibility confirmation with the onsite health care professional (either nephrologist or nurse practitioner), a research assistant obtained direct consent before completion of the TBQ.

Both studies were approved by the local Research Ethics Committee (22.069, 23.065). Direct consent was obtained from all participants. This study followed the Declaration of Helsinki.

### Survey

The TBQ multiattribute instrument (v2012, paper format), developed to specifically focus on the burden of treatment and medical follow-up among patients with multiple chronic conditions,^18^ was used to assess health-related QOL of all participants. The validity and reliability of that instrument have been previously established in numerous disease-specific populations, including adults with CKD.^19^ In addition, the questionnaire has been validated in Canada^20^ in French and English. The TBQ assesses five individual domains, including the burden related to (*1*) treatment and medication, (*2*) medical follow-up and self-monitoring, (*3*) administrative and financial difficulties, (*4*) dietary and physical restrictions, and (*5*) mental health and relationships. The 2012 version includes 15 questions (0–10 Likert scale for each, where 0 refers to “not a problem” and 10 to “big problem”), totaling to 150 points, and was offered in French or English according to the participant's preference. For each question, participants can also choose the option “does not apply.”

### Data Collection

Demographic data, such as age, sex, ethnicity, body mass index, and comorbidities, were collected at baseline. All comorbidities were collected to allow determination of the Charlson Comorbidity Index (maximum 37 points)^21^ predicting 10-year survival. The eGFR was determined using the most recent serum creatinine measurement (CKD Epidemiology Collaboration formula) at the time of survey completion. AKI and CKD staging were based on KDIGO criteria.

### Statistical Analysis

Baseline demographics data were presented as medians with interquartile ranges (IQRs) and as counts (percentages). Total QOL scores (on a possibility of 0–150, where higher scores refer to higher burden) were expressed both as medians (IQR) and means (SD) to enable comparison with other studies and for use in future health research. For score calculation, the option “does not apply” was imputed to 0, but also reported separately (Supplemental Table 1). The primary outcome of this study was the final TBQ QOL score. Scores from severe AKI survivors from the post-AKI cohort were compared with participants with CKD from the TBQ CKD cohort. Given the suspected skewed distribution of these scores, comparisons across both cohorts were made using Mann–Whitney *U* test (significance *P* < 0.05). Individual scores for each question were presented visually (forest plot) and as median (IQR). Subanalyses included the association between TBQ score and (*1*) number of daily medications, (*2*) age, (*3*) Charlson Comorbidity Index, (*4*) time from hospital discharge to survey completion, (*5*) hospital length of stay, and (6) eGFR using Spearman's rank correlation test. Exploratory analyses, using univariate linear regression, were performed to explore the association between TBQ score and relevant comorbidities or clinical parameters of interest: (*1*) AKI stage, (*2*) recent exposure to KRT, (*3*) intensive care unit (ICU) stay, (*4*) heart failure, (*5*) diabetes, and (*6*) peripheral vascular disease. All data analyses were performed using SPSS 29.0.1.0 (IBM, New York).

## Results

### Participants and Characteristics

**Figure 1:**
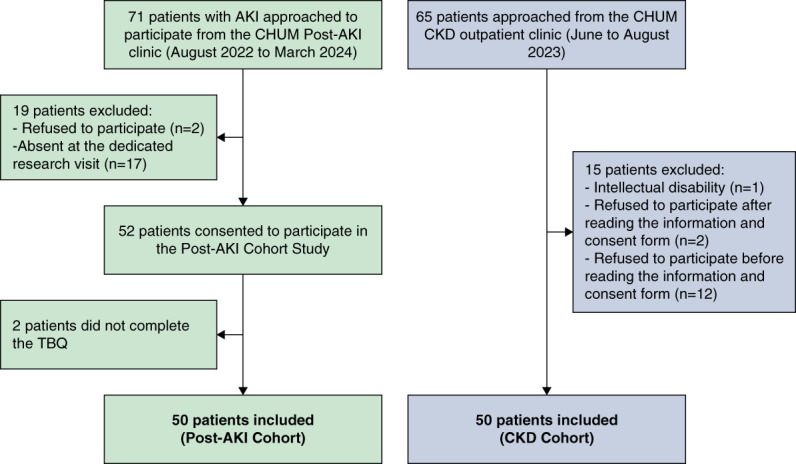
**Flow chart of all included participants per cohort (*n*=100).** CHUM, Centre hospitalier de l’Université de Montréal; TBQ, Treatment Burden Questionnaire.

Fifty-two of the 71 AKI survivors approached to participate in the Post-AKI Cohort Study between August 2022 and March 2024 gave consent. Of these participants, 50 completed the TBQ and were therefore included in this study. Of the 65 outpatients followed at the CKD renal protection clinic approached, 50 participants gave consent and completed the TBQ. The reasons for exclusion are detailed in Figure 1. As summarized in Table 1, participants from the post-AKI cohort were younger (63 years [IQR, 52–72]) than participants recruited from the CKD cohort (72 years [IQR, 62–79]), with similar sex ratios. Hypertension (80% versus 64%) and diabetes (64% versus 24%) were more frequent in the CKD cohort. However, a higher proportion of participants from the post-AKI cohort had heart failure (30% versus 14%) or peripheral vascular disease (22% versus 6%) compared with the CKD cohort. In the post-AKI cohort, half of the participants with heart failure (*n*=7) had reduced left ventricular ejection fraction (≤40%). This resulted in a Charlson Comorbidity Index higher in participants from the CKD cohort (8 [IQR, 6–9]) compared with the post-AKI cohort (5 [IQR, 2–7]). Notably, at the time of completing the TBQ, the median eGFR was 15 (IQR, 12–20) ml/min per m^2^ in the CKD cohort compared with 60 (IQR, 40–82) in the post-AKI cohort. The total number of different medications taken daily was 6 (IQR, 3–8) in the post-AKI cohort and two times higher in the CKD cohort (11 [IQR, 8–12]), representing 15 (IQR, 10–17) tablets a day. Regarding the recent AKI episode, participants from the post-AKI cohort experienced severe stage 3 AKI in most cases (84%), and 31 participants (62%) required KRT. Two thirds of these participants were hospitalized in the ICU, 21 (42%) had sepsis, and the median length of stay was 22 (IQR, 13–22) days. The median time between hospital discharge and recruitment to the Post-AKI Cohort Study where TBQ was completed was 9.7 (6.8–13.6) weeks.

**Table 1:** Baseline characteristics of participants

Variable	Post-AKI Cohort (*n*=50)	CKD Cohort (*n*=50)
Age, yr	63 (52–72)	72 (62–79)
Male sex, No. (%)	34 (68)	31 (62)
**Race, No. (%)**		
Not White	10 (20)	21 (42)
White	40 (80)	29 (58)
BMI, kg/m^2^	26.9 (24.0–30.9)	28.6 (24.0–32.0)
Charlson Comorbidity Index	5 (2–7)	8 (6–9)
**Comorbidities, No. (%)**		
Hypertension	32 (64)	40 (80)
Diabetes mellitus	12 (24)	32 (64)
Cardiovascular disease	15 (30)	14 (28)
Heart failure	15 (30)	7 (14)
Peripheral vascular disease	11 (22)	3 (6)
Stroke	8 (16)	6 (12)
Chronic obstructive pulmonary disease	9 (18)	8 (16)
Cancer	14 (28)	13 (26)
*Localized*	11 (22)	10 (20)
*Metastatic*	3 (6)	3 (6)
**Kidney function at the time of survey completion**		
CKD KDIGO stage, No. (%)		
*Stage 3*	16 (32)	0
*Stage 4*	8 (16)	29 (58)
*Stage 5*	0	21 (42)
eGFR, ml/min per 1.73m^2^	60 (40–82)	15 (12–20)
**AKI episode characteristics**		N/A
AKI KDIGO stage, No. (%)		
*Stage 1*	0	
*Stage 2*	8 (16)	
*Stage 3*	42 (84)	
KRT status (any modality), No. (%)	31 (62)	
*Continuous KRT*	18 (36)	
*Intermittent KRT*	28 (56)	
AKI etiology, No. (%)		
*Prerenal or functional*	6 (12)	
*Ischemic ATN*	32 (64)	
*Nonischemic ATN*	17 (34)	
*Other*	1 (2)	
ICU stay, No. (%)	32 (64)	
Hospital length of stay, d	22 (13–32)	
Hospitalization unit, No. (%)		
*Medical*	30 (60)	
*Surgical*	20 (50)	
Sepsis status, No. (%)	21 (42)	
No. of active oral medications	6 (3–8)	11 (8–12)
No. of tablets taken daily	8 (4–10)	15 (10–17)

Kidney function: last serum creatinine measurement available at the time of the Treatment Burden Questionnaire survey was used to determine eGFR and CKD KDIGO stages. AKI etiology: more than one etiology possible. Functional AKI included prerenal azotemia, cardiorenal syndrome, and hepatorenal syndrome. Nonischemic acute tubular necrosis included nephrotoxicity and nonhypotensive septic-associated AKI. Other refers to obstructive AKI (*n*=1). Surgical units included cardiac or vascular surgery (*n*=8), GI or hepatobiliary surgery (*n*=10), and urology (*n*=2). ATN, acute tubular necrosis; BMI, body mass index; GI, gastrointestinal; ICU, intensive care unit; KDIGO, Kidney Disease Improving Global Outcomes.

### TBQ Scores

**Figure 2:**
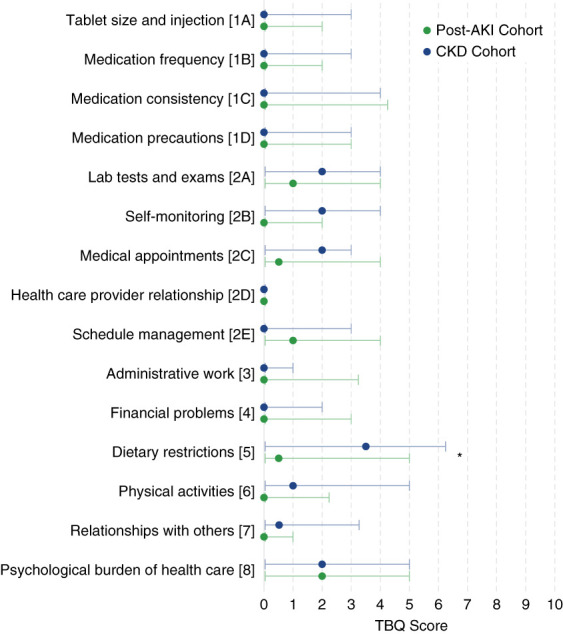
**Forest plot of TBQ results according to both cohorts.** Representing median (dots) and IQR (error bars) of all questions. Higher TBQ score means higher burden. Only Q5 (dietary restrictions) was statistically significant when comparing TBQ results between the CKD cohort and post-AKI cohort (*P* = 0.037, Mann–Whitney *U* test). IQR, interquartile range.

The median TBQ final score representing health-related QOL was 19.0 (IQR, 6.5–43.8) in the post-AKI cohort and 25.0 (12.8–43.0) in the CKD cohort and did not differ significantly (*P* = 0.45). The mean score was 27.0 (SD, 23.6) and 28.2 (SD, 19.3), respectively. Figure 2 reports the score for each question, stratified by cohort (Supplemental Table 1). Participants from the CKD cohort reported greater effect regarding dietary restrictions (Q5, *P* = 0.037). No other statistically significant differences were observed between the two cohorts, including in treatment and medication-related burden (Q1A, 1B, 1C, and 1D) or medical follow-up (Q2A, 2C, 2D, and 2E). There was a trend toward greater effects on QOL regarding self-monitoring (Q2B, *P* = 0.065) and relationships with others (Q7, *P* = 0.135) in participants from the CKD cohort. Most of the participants (77%) reported no to minimal relationship difficulties with their health care professionals, whereas 33% of them reported a score of 5 or higher, representing at least a moderate effect, when asked if “the need for medical health care on a regular basis reminds me of my health problems,” which was similar in both cohorts.

### Subanalyses

The correlation between participant age and TBQ score was negatively correlated (Spearman's rank: −0.23 [−0.41 to −0.03], *P* = 0.02), especially in the CKD cohort (−0.51 [−0.70 to −0.26], *P* < 0.001), meaning older participants reported a lower effect on health-related QOL. Likewise, a negative correlation was observed between Charlson Comorbidity Index and TBQ score in the CKD cohort (−0.32 [−0.60 to −0.10], *P* = 0.01), but not in post-AKI participants (−0.18 [−0.44 to 0.11], *P* = 0.21). A negative correlation was also observed between eGFR measurement and TBQ score (−0.20 [−0.38 to 0.01], *P* = 0.049), especially in the CKD cohort (−0.31 [−0.54 to −0.02], *P* = 0.03), meaning lower eGFR correlates with lower reported health-related QOL. No correlation was observed when considering the medication burden represented by the number of tablets taken daily (−0.05 [−0.25 to 0.16], *P* = 0.64). In the post-AKI cohort, there was no correlation observed between TBQ score and hospital length of stay (0.09 [−0.21 to 0.36], *P* = 0.55) or the time between hospital discharge and TBQ completion (−0.12 [−0.40 to 0.17], *P* = 0.40; Supplemental Figure 1).

In the post-AKI cohort, TBQ score was not associated with KDIGO AKI stage (*P* = 0.43), recent exposure to KRT (*P* = 0.57), or ICU admission (*P* = 0.95). Regarding specific comorbidities of interest, TBQ score was not associated with heart failure (post-AKI cohort, *P* = 0.33; CKD cohort, *P* = 0.34) or diabetes (post-AKI cohort, *P* = 0.52; CKD cohort, *P* = 0.87), but was associated with the presence of peripheral vascular disease (post-AKI cohort, *P* = 0.01; CKD cohort, *P* = 0.22).

## Discussion

In this study, our first aim was to explore the self-reported health-related QOL and treatment burden in patients who have had severe AKI when considering patient-oriented care. We showed that severe AKI survivors had similar TBQ results to patients living with advanced CKD. Despite being younger, presenting fewer comorbidities, and taking fewer medications, the self-reported health burden was remarkably comparable in both cohorts. Interestingly, we identified a negative correlation between age, number of comorbidities, and residual kidney function with TBQ score, especially in the advanced CKD cohort. On the contrary, in AKI survivors, there was no clear association between the severity of AKI episode, including recent exposure to KRT, and QOL.

Advanced CKD, including patients undergoing KRT, is complicated by diverse metabolic anomalies in which particular dietary recommendations, including restrictions, have long been considered essential,^22^ despite negatively affecting the overall QOL.^23^ In this study, we confirm that patients with CKD followed in a dedicated nephrology clinic and receiving standard recommendations regarding CKD diet reported these dietary restrictions as a substantial burden on their QOL. As expected, AKI survivors with relatively preserved eGFR did not report such an effect.

Identification of the burden of symptoms and QOL for patients living with advanced CKD has been considered a priority by clinicians and patients themselves. Some symptoms and restrictions reported by patients with CKD can be mitigated by changes in clinical management,^24,25^ including the psychological stress of living with a chronic disease, through adequate support and multidisciplinary and patient-centered care.^6,16^ Such an approach has been validated in various nephrology populations through the Standardized Outcomes in Nephrology Initiative, developing shared priorities relevant to not only health care professionals and researchers, but also patients and their families.^26^

In patients with AKI, a study using data from the Veterans Affairs/National Institutes of Health Acute Renal Failure Trial reported that severe AKI survivors (all exposed to KRT) had health-related QOL severely compromised at 60-day follow-up, where hospital length of stay and comorbidities were major predictors of lower QOL.^7^ In another study involving participants having experienced an AKI episode, 84% of them reported that this episode was “very” or “extremely” impactful on their physical and emotional health.^9^ That study, based on an anonymous survey distributed to members from the American Association of Kidney Patients, did not explore the association between QOL results and objective medical measures as we reported here. Another study using the Short Form-36 questionnaire in postoperative ICU survivors and distributed at 6-month follow-up also showed lower QOL in those who had an AKI event.^10^ Using a dedicated post-AKI follow-up clinic, the current study confirms the low self-reported QOL in severe AKI survivors and presents enough granularity to explore potential associations with major objective measures, such as AKI severity, residual eGFR, hospital length of stay, and particular comorbidities.

In our cohort, most AKI of the survivors had experienced prolonged hospitalization, ICU admission, numerous complications, and even acute KRT initiation. Such traumatic experiences may affect the self-reported QOL once discharged, sometimes referred to as posthospitalization syndrome. In this study, despite fewer comorbidities, medications, and overall medical care, AKI survivors reported a comparable QOL with patients with advanced CKD, known to have a significantly lower QOL than the general population.^1,6^ Importantly, self-reported health-related QOL did not positively correlate with standard morbidity risk factors, such as age, AKI severity, or hospital length of stay. The substantial effect of an AKI episode observed on health-related QOL may be partially attributed to the presence of coexisting comorbidities, such as heart failure,^27^ but also as part of the overall experience of hospitalization in which occurred a sudden and unexpected need for close medical follow-up after hospital discharge. This new sick status is often accompanied by new medications and additional restrictions (*e.g*., financial, dietary, physical, and work) as previously reported.^9^ In comparison, people experiencing a more progressive course of disease, such as progressive CKD, may have already adjusted to this situation and its associated burden on their daily life.

The current study was not designed to specifically capture, in participants from the post-AKI cohort, whether the burden reported on QOL was attributed to the AKI episode and associated care or related to the recent and complex hospitalization. Though, no clear correlation was observed regarding Charlson Comorbidity Index, hospital length of stay, or follow-up time since discharge, arguing in disfavor of a posthospitalization syndrome. Regardless of the final explanation, similar to the care offered to patients living with CKD, this study demonstrated the importance of considering the QOL during the follow-up of AKI survivors.

Strengths of this study include the use of a comparison group (advanced CKD cohort) known to have low health-related QOL. In addition, the granularity of all variables collected allowed us to perform subanalyses on major variables that affect patients' QOL, including KRT exposure, age, hospital length of stay, particular comorbidities, and medication burden. Our findings present some limitations. First, only participants from a single center were included. Participants had to be referred to the post-AKI clinic and to survive until the planned follow-up to be approached, which may limit generalizability. Participants were recruited from a large urban academic center which benefits from the nephrology expertise and resources to follow such patients, which may not be generalizable to all centers. GFR was estimated using serum creatinine, which presents some limitations, especially in the post-AKI cohort, because prolonged hospitalizations are associated with muscle wasting, sarcopenia, and lower creatinine production, which might have overestimated the true kidney function in this population.^28^ In addition, the sample size was limited, especially to infer robust conclusions regarding exploratory analyses. In this study, the Charlson Comorbidity Index, using binomial data on the presence or absence of specific comorbidities, may lack precision to correctly quantify the burden of these comorbidities in such complex patients. Finally, the TBQ has the advantage of being easy to perform, understand, and analyze, but may lack some comprehensiveness because of its short format. Nevertheless, this study is one of the first to focus on health-related QOL in severe AKI survivors using a well-known comparison group and provides important information on that neglected nephrology population. Such data, despite being preliminary, highlight the need to further consider a holistic patient-centered approach when offering post-AKI care.

In summary, this study enlightens that an AKI episode may have substantial consequences on health-related QOL weeks after hospital discharge. This preliminary study shows that severe AKI survivors have a self-reported QOL comparable with the low QOL of people living with advanced CKD. This highlights the need to raise awareness on the effect of high-grade AKI on patient's QOL. However, how to integrate that information into medical practice as part of a patient-centered approach tailored to the needs of this particular population when offering post-AKI care requires further research.

## Supplementary Material

SUPPLEMENTARY MATERIAL

## Data Availability

Partial restrictions to the data and/or materials apply. The data that support the findings of this study are available on request from the corresponding author. The data are not publicly available due to privacy or ethical restrictions.

## Supplemental Material

Self-reported Burden and Health-related QOL in AKI Survivors compared with Patients with Advanced CKD

Supplemental Table 1. Median TBQ score per question categorized by cohort.

Supplemental Figure 1A. Correlation between TBQ score and age (years).

Supplemental Figure 1B. Correlation between TBQ score and total number of tablets per day.

Supplemental Figure 1C. Correlation between TBQ score and Charlson Comorbidity Index.

Supplemental Figure 1D. Correlation between TBQ score and time between hospital discharge and TBQ.

Supplemental Figure 1E. Correlation between TBQ score and hospital length of stay.

Supplemental Figure 1F. Correlation between TBQ score and eGFR at the time of survey completion.
