# Supplementary material for: Self-Reported Burden and Health-Related Quality of Life in Acute Kidney Injury Survivors Compared with Patients with Advanced CKD
Source: Kidney360. 2025 Jan 17;6(5):720–7. doi: 10.34067/KID.0000000707 (PMC12136637; doi:10.34067/KID.0000000707)
Supplement: SUPPLEMENTARY MATERIAL [file kidney360-6-720-s001.pdf]

ASN American Society of Nephrology | JASN Journal of the American Society of Nephrology | KIDNEY360 Accessing Our World From Every Angle | CJASN Clinical Journal of the American Society of Nephrology

ASN Journal Disclosure Form

As per ASN journal policy, I have disclosed any financial relationships or commitments I have held in the past 36 months as included below. I have listed my Current Employer below to indicate there is a relationship requiring disclosure. If no relationship exists, my Current Employer is not listed.

J. Cote reports the following:
Employer: CHUM; and Other Interests or Relationships: Member - Canadian Society of Nephrology; Nephrology Consultant - CHUM (Montreal).

I understand that the information above will be published within the journal article, if accepted, and that failure to comply and/or to accurately and completely report the potential financial conflicts of interest could lead to the following: 1) Prior to publication, article rejection, or 2) Post-publication, sanctions ranging from, but not limited to, issuing a correction, reporting the inaccurate information to the authors' institution, banning authors from submitting work to ASN journals for varying lengths of time, and/or retraction of the published work.

Name: Jean Maxime Cote
Manuscript ID: K360-2024-000712R1
Manuscript Title: Self-reported Burden and Health-related Quality of Life in Acute Kidney Injury Survivors compared to Patients with Advanced CKD
Date of Completion: December 2, 2024
Disclosure Updated Date: December 2, 2024

ASN American Society of Nephrology | JASN Journal of the American Society of Nephrology | KIDNEY360 Accessing Our World From Every Angle | CJASN Clinical Journal of the American Society of Nephrology

ASN Journal Disclosure Form

As per ASN journal policy, I have disclosed any financial relationships or commitments I have held in the past 36 months as included below. I have listed my Current Employer below to indicate there is a relationship requiring disclosure. If no relationship exists, my Current Employer is not listed.

I. Ethier reports the following:
Research Funding: Co-local investigator for DIALIZE-Outcomes study, funded by AstraZeneca (study now closed); and Other Interests or Relationships: Member: Quality Improvement & Implementation Sciences and Sustainable Nephrology Action Planning committees of Canadian Society of Nephrology; International Society of Nephrology - Emerging Leaders Program cohort 2; Néphrologie Verte group of Société Francophone de Néphrologie Dialyse et Transplantation; all memberships unpaid and voluntary. Supported by grant from Fonds de Recherche du Québec Santé (FRQS) under the Clinical Research Scholars - Junior 1 program.

I understand that the information above will be published within the journal article, if accepted, and that failure to comply and/or to accurately and completely report the potential financial conflicts of interest could lead to the following: 1) Prior to publication, article rejection, or 2) Post-publication, sanctions ranging from, but not limited to, issuing a correction, reporting the inaccurate information to the authors' institution, banning authors from submitting work to ASN journals for varying lengths of time, and/or retraction of the published work.

Name: Isabelle Ethier
Manuscript ID: K360-2024-000712R1
Manuscript Title: Self-reported Burden and Health-related Quality of Life in Acute Kidney Injury Survivors compared to Patients with Advanced CKD
Date of Completion: November 6, 2024
Disclosure Updated Date: November 6, 2024

ASN American Society of Nephrology
JASN Journal of the American Society of Nephrology
KIDNEY360 Accessing Our World From Every Angle
CJASN Clinical Journal of the American Society of Nephrology

ASN Journal Disclosure Form

As per ASN journal policy, I have disclosed any financial relationships or commitments I have held in the past 36 months as included below. I have listed my Current Employer below to indicate there is a relationship requiring disclosure. If no relationship exists, my Current Employer is not listed.

F. Huang reports the following:
Research Funding: Université de Montréal - "Programme PREMIER"

I understand that the information above will be published within the journal article, if accepted, and that failure to comply and/or to accurately and completely report the potential financial conflicts of interest could lead to the following: 1) Prior to publication, article rejection, or 2) Post-publication, sanctions ranging from, but not limited to, issuing a correction, reporting the inaccurate information to the authors' institution, banning authors from submitting work to ASN journals for varying lengths of time, and/or retraction of the published work.

Name: Felix Huang
Manuscript ID: K360-2024-000712R1
Manuscript Title: Self-reported Burden and Health-related Quality of Life in Acute Kidney Injury Survivors compared to Patients with Advanced CKD
Date of Completion: November 9, 2024
Disclosure Updated Date: November 9, 2024

ASN American Society of Nephrology JASN Journal of the American Society of Nephrology KIDNEY360 Accessing Our World From Every Angle CJASN Clinical Journal of the American Society of Nephrology

ASN Journal Disclosure Form

As per ASN journal policy, I have disclosed any financial relationships or commitments I have held in the past 36 months as included below. I have listed my Current Employer below to indicate there is a relationship requiring disclosure. If no relationship exists, my Current Employer is not listed.

S. Silver reports the following:
Employer: Queen's University; Consultancy: Astra Zeneca; Research Funding: CSL Behring, Boehringer Ingelheim; Honoraria: Baxter, Otsuka, Novo Nordisk, Boehringer Ingelheim, Bayer; and Advisory or Leadership Role: Canadian Society of Nephrology Board Member.

I understand that the information above will be published within the journal article, if accepted, and that failure to comply and/or to accurately and completely report the potential financial conflicts of interest could lead to the following: 1) Prior to publication, article rejection, or 2) Post-publication, sanctions ranging from, but not limited to, issuing a correction, reporting the inaccurate information to the authors' institution, banning authors from submitting work to ASN journals for varying lengths of time, and/or retraction of the published work.

Name: Samuel A. Silver
Manuscript ID: K360-2024-000712R1
Manuscript Title: Self-reported Burden and Health-related Quality of Life in Acute Kidney Injury Survivors compared to Patients with Advanced CKD."
Date of Completion: November 6, 2024
Disclosure Updated Date: October 22, 2024

ASN American Society of Nephrology · JASN Journal of the American Society of Nephrology · KIDNEY360 Accessing Our World From Every Angle · CJASN Clinical Journal of the American Society of Nephrology

ASN Journal Disclosure Form

As per ASN journal policy, I have disclosed any financial relationships or commitments I have held in the past 36 months as included below. I have listed my Current Employer below to indicate there is a relationship requiring disclosure. If no relationship exists, my Current Employer is not listed.

I. Vaillant reports the following:
Employer: CHUM (centre hospitalier de l'université de Montréal); and Consultancy: Otsuka (comité consultatif régional juin 2024).

I understand that the information above will be published within the journal article, if accepted, and that failure to comply and/or to accurately and completely report the potential financial conflicts of interest could lead to the following: 1) Prior to publication, article rejection, or 2) Post-publication, sanctions ranging from, but not limited to, issuing a correction, reporting the inaccurate information to the authors' institution, banning authors from submitting work to ASN journals for varying lengths of time, and/or retraction of the published work.

Name: Isabelle Vaillant
Manuscript ID: K360-2024-000712R1
Manuscript Title: Self-reported Burden and Health-related Quality of Life in Acute Kidney Injury Survivors compared to Patients with Advanced CKD
Date of Completion: January 13, 2025
Disclosure Updated Date: January 13, 2025
