# Supplementary material for: Self-Reported Burden and Health-Related Quality of Life in Acute Kidney Injury Survivors Compared with Patients with Advanced CKD
Source: Kidney360. 2025 Jan 17;6(5):720–7. doi: 10.34067/KID.0000000707 (PMC12136637; doi:10.34067/KID.0000000707)
Supplement: SUPPLEMENTARY MATERIAL [file kidney360-6-720-s002.pdf]

Supplementary Materials

Self-reported Burden and Health-related Quality of Life in Acute Kidney Injury Survivors compared to Patients with Advanced CKD

Page 2: **Table S1**. Median TBQ score per question categorized by cohort

Page 3: **Figure S1A**. Correlation between TBQ Score and Age (years)

Page 4: **Figure S1B**. Correlation between TBQ Score and total number of tablets per day

Page 5: **Figure S1C**. Correlation between TBQ Score and Charlson Comorbidity Index

Page 6: **Figure S1D**. Correlation between TBQ Score and Time between hospital discharge and TBQ

Page 7: **Figure S1E**. Correlation between TBQ Score and Hospital length of stay

Page 8: **Figure S1F**. Correlation between TBQ Score and eGFR at the time of survey completion

Table S1. Median TBQ score per question categorized by cohort (Median + IQR)

TBQ question/ domain	Post-AKI Cohort (n = 50)	CKD Cohort (n = 50)	p-value[1]
(1A) Annoyances caused by pills/injections	0 (0-2)	0 (0-3)	0.255
Does not apply (%)	6 (12)	0	
(1B) Daily frequency of medication burden	0 (0-2)	0 (0-3)	0.421
Does not apply (%)	5 (10)	0	
(1C) Efforts to not to forget medication burden	0 (0-4.25)	0 (0-4)	1.0
Does not apply (%)	3 (6)	1 (2)	
(1D) Precautions when taking medication burden	0 (0-3)	0 (0-3)	0.760
Does not apply (%)	6 (12)	5 (10)	
(2A) Lab tests and other exams burden	1 (0-4)	2 (0-4)	0.925
Does not apply (%)	2 (4)	1 (2)	
(2B) Self-monitoring burden	0 (0-2)	2 (0-4)	0.065
Does not apply (%)	11 (22)	1 (2)	
(2C) Inconveniences of doctor appointments	0.5 (0-4)	2 (0-3)	0.721
Does not apply (%)	1 (2)	0	
(2D) Relationship difficulties with healthcare providers	0 (0-0)	0 (0-0)	0.745
Does not apply (%)	8 (16)	3 (6)	
(2E) Medical appointments arrangement burden	1 (0-4)	0 (0-3)	0.509
Does not apply (%)	0	2 (4)	
(3) Administrative burden related to healthcare	0 (0-3.25)	0 (0-1)	0.150
Does not apply (%)	8 (16)	13 (26)	
(4) Financial burden related to healthcare	0 (0-3)	0 (0-2)	0.866
Does not apply (%)	6 (12)	13 (26)	
(5) Dietary changes burden	0.5 (0-5)	3.5 (0-6.25)	0.037
Does not apply (%)	11 (22)	2 (4)	
(6) Physical activity recommendations burden	0 (0-2.25)	1 (0-5)	0.267
Does not apply (%)	9 (18)	6 (12)	
(7) Difficulties in relationships with others	0 (0-1)	0.5 (0-3.25)	0.135
Does not apply (%)	5 (10)	3 (6)	
(8) Healthcare reminds me of my health problems	2 (0-5)	2 (0-5)	0.577
Does not apply (%)	4 (8)	2 (4)	
**Total score**	**19.0 (6.5-43.75)**	**25.0 (12.75-43.0)**	**0.448**

[1] Mann-Whitney U Test (significance p=0.05)
Treatment Burden Questionnaire (TBQ): Higher score is associated with higher burden (0-10 Likert Scale). 'Does not apply' were converted to '0' for all statistical analysis.

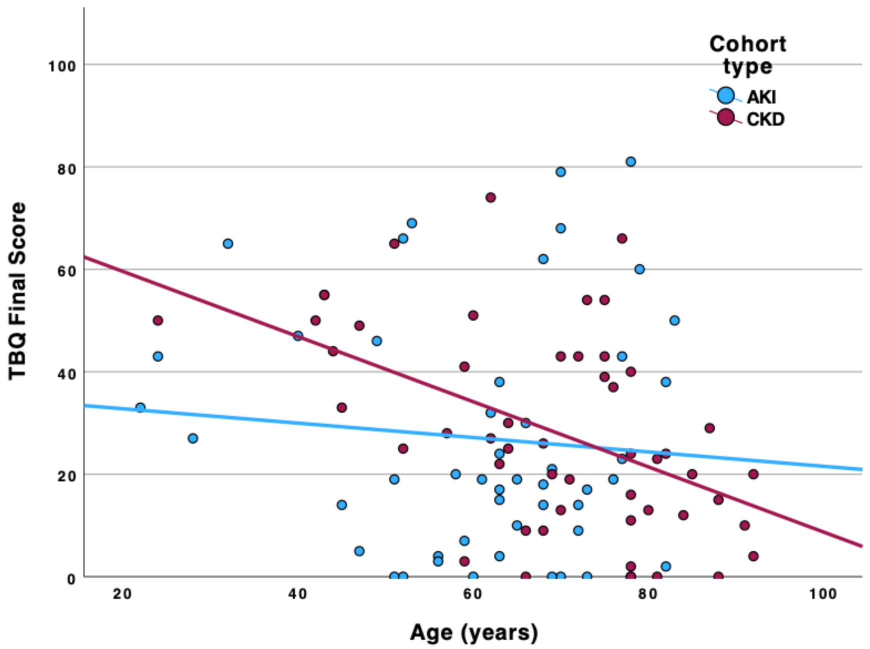

Figure S1A. Correlation between TBQ Score and Age (years)

Spearman's rho TOTAL: -0.228 [-0.411; -0.027] (p=0.023)

Spearman's rho AKI: -0.047 [-0.329; 0.243] (p=0.747)

Spearman's rho CKD: -0.512 [-0.696; -0.264] (p<0.001)

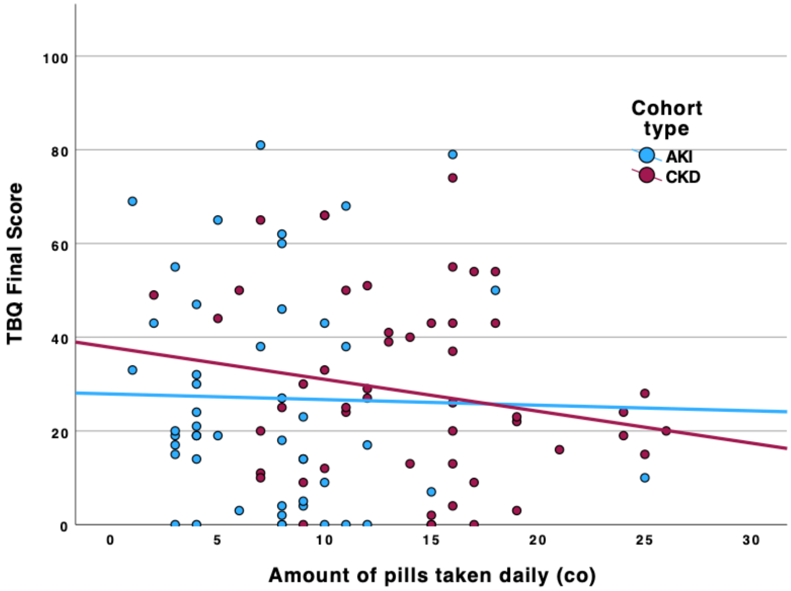

Figure S1B. Correlation between TBQ Score and total number of tablets per day

Spearman's rho TOTAL : -0.048 [-0.247; 0.156] (p=0.638)

Spearman's rho AKI: -0.130 [-0.401; 0.162] (p=0.367)

Spearman's rho CKD: -0.162 [-0.429; 0.130] (p=0.260)

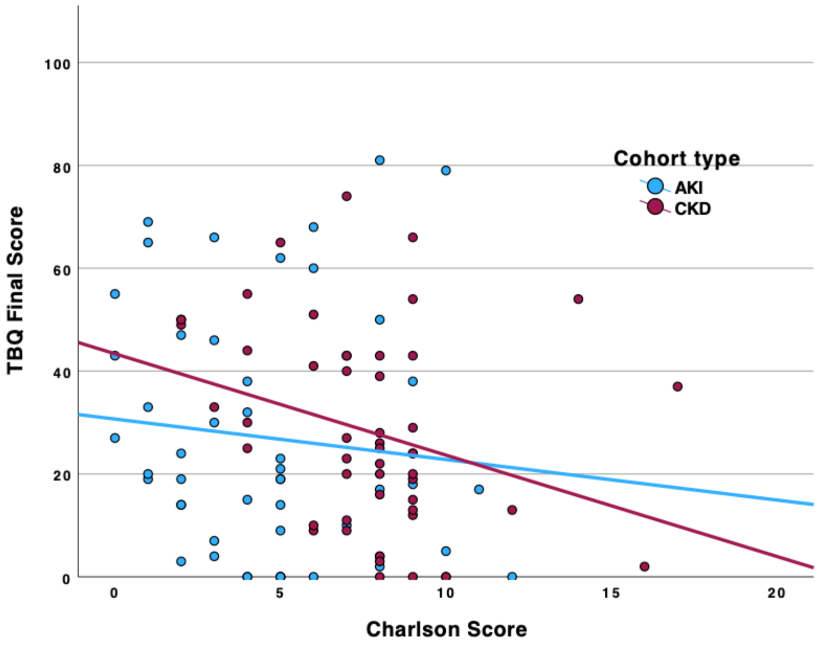

Figure S1C. Correlation between TBQ Score and Charlson Comorbidity Index

Spearman's rho TOTAL: -0.192 [-0.379; 0.010] (p=0.055)

Spearman's rho AKI: -0.181 [-0.444; 0.110] (p=0.207)

Spearman's rho CKD: -0.317 [-0.595; -0.096] (p=0.008)

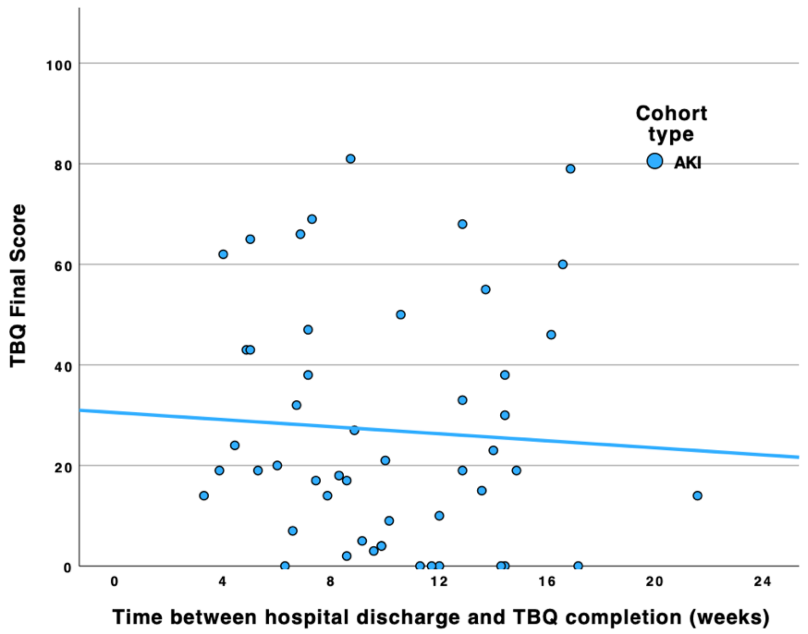

Figure S1D. Correlation between TBQ Score and Time between hospital discharge and TBQ completion (weeks) (Only AKI Survivors)
Spearman's rho AKI: -0.122 [-0.395; 0.170] (p=0.397)

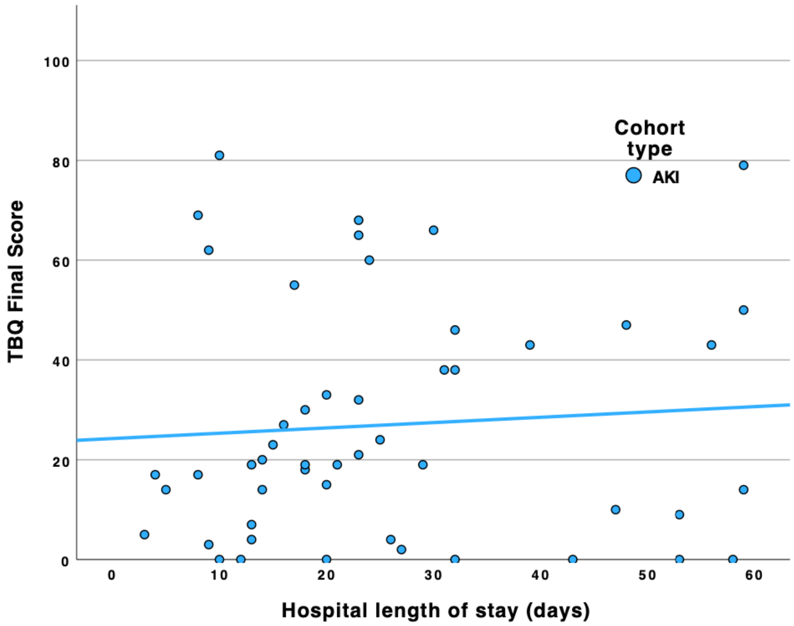

Figure S1E. Correlation between TBQ Score and Hospital length of stay (days) (Only AKI Survivors)
Spearman's rho AKI: 0.087 [-0.205; 0.364] (p=0.549)

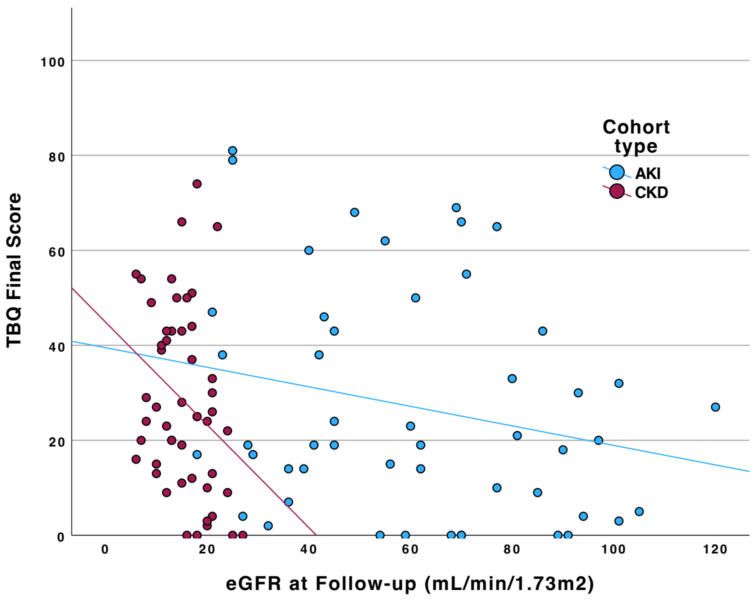

Figure S1F. Correlation between TBQ Score and eGFR at the time of survey completion

Spearman's rho TOTAL: -0.197 [-0.384; 0.005] (p=0.049)

Spearman's rho AKI: -0.190 [-0.451; 0.102] (p=0.187)

Spearman's rho CKD: -0.302 [-0.541; -0.017] (p=0.033)
